# MDMA Abuse in Relation to MicroRNA Variation in Human Brain Ventral Tegmental Area and Nucleus Accumbens

**DOI:** 10.22037/ijpr.2019.15097.12874

**Published:** 2019

**Authors:** Goksun Demirel, Esra Guzel, Chad J Creighton, Yeter Erol Ozturk, Ceyhun Kucuk, Hızır Asliyuksek, Türkan Yurdun

**Affiliations:** a *Department of Pharmaceutical Toxicology, Faculty of Pharmacy, Biruni University, Istanbul, Turkey. *; b *Department of Pharmaceutical Toxicology, Faculty of Pharmacy, Çukurova University, Adana, Turkey. *; c *Department of Molecular Biology and Genetics, Institute of Health Sciences, University of Health Sciences, Istanbul, Turkey. *; d *Department of Medicine, Baylor College of Medicine, Houston, Texas. *; e *Dan L. Duncan Cancer Center Division of Biostatistics, Houston, Texas. *; f *Chemistry Department, Council of Forensic Medicine, Istanbul, Turkey. *; g *Council of Forensic Medicine, Istanbul, Turkey. *; h *Department of Pharmaceutical Toxicology, Faculty of Pharmacy, Marmara University, Istanbul, Turkey.*; i *G D and E G. These authors contributed equally to this work.*

**Keywords:** MicroRNA, 3, 4-methylenedioxymethamphetamine, Ventral Tegmental Area, Nucleus Accumbens, Microarray research

## Abstract

3,4-methylenedioxymethamphetamine (MDMA) is one of the most widespread illegal drugs, that have been used particularly by young people in the 15-34 age group. MicroRNAs (miRNAs) are endogenously synthesized, non-coding, and small RNAs that post-transcriptionally regulate their target genes’ expression by inhibiting protein translation or degradation. miRNAs are increasingly implicated in drug-related gene expressions and functions. Notably, there are no reports of miRNA variation in the human brain in MDMA abuse. We here present a miRNA profiling study – the first such study, to the best of our knowledge – into the post-mortem human brains of a sample of people with MDMA abuse, along with non-drug dependent controls. The miRNA profiling of nucleus accumbens (NAc) and ventral tegmental areas (VTA) was performed by microarray analysis. Subsequently, two candidate miRNA putative biomarkers were selected according to significant regional differential expression (miR-1202 and miR-7975), using quantitative reverse-transcription PCR (qRT-PCR). We showed that the expression level of miR-7975 was significantly lower in the VTA regions of the 30 MDMA users, as compared with the 30 control samples. Another significantly deregulated miR-1202 was down-regulated in the NAc regions of 30 MDMA samples in comparison to the control samples. Alteration of these miRNAs can potentially serve as novel biomarkers for MDMA abuse, and warrant further research in independent and larger samples of patients.

## Introduction

MDM(3,4methylenedioxymethamphetamine), commonly known as ‘ecstasy’ or ‘molly’, is an illicit, synthetic drug which causes serotonergic neurotoxicity in the brain (1). Prosocial behavior and empathogenic feelings are boosted by MDMA’s effect on emotional and cognitive empathy processes. MDMA is considered to have therapeutic effects in psychotherapy; however, high-dose MDMA use has been associated with multiple complications such as persisting cognitive deficits and panic attacks (2-4), and can also lead to drug addiction, toxicity, or death (5, 6). MDMA, the most frequently-used amphetamine-type stimulant, causes the release of serotonin, dopamine, and norepinephrine in the parts of the brain that are targeted by other addictive drugs (7). Addictive drugs target the mesocorticolimbic dopamine (DA) system in the addiction center of the brain, including the ventral tegmental area (VTA), prefrontal cortex, and nucleus accumbens (NAc) (8, 9). The VTA, which contains the largest group of dopamine neurons, plays an important role in reward-related and goal-directed behaviors such as cognitive and emotional processes (10-12). The NAc receives dopaminergic inputs from the VTA and has been associated with control of behavior by drug-related stimuli and with relapse. (13). Hence, the VTA and NAc are central components of the brain’s reward system, and also play a major role in the response to drugs abuse (14, 15).

The latest data suggests that MDMA has become one of the most popularly used illegal drugs in Europe (9.3 million males and 4.7 million females). The European Drug Report has estimated that the availability and use of high-dose MDMA tablets is increasing, and this trend is set to continue. The same report emphasized that the number of people dying from MDMA use has significantly increased in recent years (16). Therefore, it is necessary to understand the molecular mechanism underlying the progression of MDMA abuse in order to find specific biomarkers for therapeutic approaches. There is an increasing body of evidence indicating that microRNAs (miRNAs) are associated with the regulation of drug-induced pathways, including the improvement of drug-addiction behaviors (17). 

miRNAs are approximately 18-22 bp long post-translational regulators that target mRNAs to repress post-transcriptional gene expression by binding to complementary sequences in the 3’UTR (18, 19). Studies have demonstrated that miRNAs play a significant role in the activity of the brain’s regions in relation to drug addiction, including the NAc, VTA and other regions of the brain’s reward pathways (20).

Many studies have demonstrated that miRNA’s expression levels are altered by drugs such as heroin (21), cocaine (22), nicotine (23), and opioids (24). However, miRNA expression profiles in postmortem human MDMA users have been never shown before. Therefore, it is of importance to study miRNA profiling specifically in the brain regions associated with MDMA seeking. 

We aimed to determine the miRNA expression profiles of the NAc and VTA regions in MDMA users’ postmortem brain tissues and matched controls in this study. We found miR-1202 and miR-7975, which are significantly down-regulated in the brain samples with MDMA, can be used as candidate biomarkers for treatment approaches to MDMA-seeking behavior.

## Experimental

*Collection of Postmortem Brain Samples*

This study has been reviewed and approved by an institutional review board of Marmara University Faculty of Medicine (IRB No: 09.2016.376). Postmortem human brain tissues consisting of the nucleus accumbens and ventral tegmental areas of 30 positive MDMA-users and 30 control samples were collected from the Morgue Department of the Council of Forensic Medicine in Istanbul, Turkey. Informed consent was obtained from family members of the postmortem individuals prior to autopsy, in accordance with the standards established by the Council of Forensic Medicine.

Blood tests were used to detect the presence of MDMA in those subjects included in this study. The drug-free control group was selected from postmortem individuals who had a negative result for all drugs, and no history of substance use prior to death. In both groups, the following cases were excluded: significant head injury, the presence of an infectious disease (e.g HIV/AIDS, hepatitis B or C), chronic disease, or a history of mental disorders. The brains were removed during autopsy, and tissue samples were immediately collected from the VTA and the NAc, then were flash-frozen in liquid nitrogen. The frozen tissues were individually kept in microcentrifuge tubes at -80 °C until RNA extraction.

*Screening of Blood Samples in Postmortem Individuals*

All blood samples were screened by general unknown screening. In this general method, the analysis of drugs was accomplished by liquid chromatography, coupled with tandem mass spectrometry (LC– MS/MS) and gas chromatography-mass spectrometry (GC–MS) techniques. GC– MS analyses were carried out using a Shimadzu 2010 QP GC–MS instrument and a Restek RTX-5-MS (30m 0.25mm 0.25mm; 5% phenylmethylsiloxane) column was used for chromatographic separations. The mass detector was operated in electronic impact mode at 70 eV and the mass spectra were scanned at m/z 60–550 amu. The injection volume was 1 µL in splitless mode. LC–MS/MS analyses were carried out using an Agilent 6460 triple quadruple tandem mass spectrometer instrument and Agilent Poroshell C18 (dimensions 150 mm 2.7 mm 4.6 mm) column for chromatographic separations. Tandem mass spectrometry (MS/MS) detection was performed using electrospray positive and negative ionization in MRM mode, with an injection volume of 10 µL. Many reports have shown that a toxic or even fatal dose entails blood levels ranging from 0.5 to 10 mg/L in most cases (25).

*Total RNA Extraction*

NAc and VTA brain tissues were blended in 1 mL TRIzol reagent (Invitrogen, USA) per 50-100 mg of tissue. Total RNA was extracted as per the manufacturer’s instructions. Total RNA concentrations and purities of the samples were quantified by spectrophotometry using a NanoDrop ND-2000 system (Thermo Fisher Scientific, Inc., Wilmington, DE). The RNA Integrity Number (RIN) was measured by the Bioanalyzer 2100 (Agilent Technologies, USA).

*miRNA Microarray and Data Analysis*

miRNA expression profiling of the NAc and VTA regions of eight postmortem brain samples (four of them from MDMA users and four of them from the control group) were performed using Agilent 8X60K Human miRNA V21.0. An Agilent miRNA microarray slide contains 2,549 probes, including control probes, according to the latest version of Sanger miRBase (Release 21.0). All of the related reagents were purchased from Agilent Inc. 50 ng of total RNA from four MDMA users and four control brain tissues were labeled with Cy3, using the “Agilent miRNA Labeling and Hybridisation Kit”, following the protocol recommended by the manufacturers. Each labeled RNA sample was hybridized onto an Agilent 8X60K Human miRNA microarray V21 chip and incubated at 55 °C for 20 h. The chip was washed after hybridization and immediately scanned by an Agilent Microarray Scanner with Surescan High Resolution Technology (Agilent Technologies, Santa Clara, CA). Feature Extraction v10.7.3.1 (Agilent Technologies) software was used to analyze the scanned images. Bioconductor software, a normalization tool for microarray data, was used on the raw data via R version 2.15 quantile normalization. Log transformed data was used to calculate fold change and P value for each sample. Potential candidate miRNAs with *p *< 0.01 and 1.4-fold change were selected for further validation by quantitative reverse transcription-polymerase chain reaction (qRT-PCR). The microarray data were then deposited into the NCBI Gene Expression Omnibus (GEO, Accession number GSE104315).

*cDNA Synthesis and qRT-PCR*

To validate the differential expression of miR-1202 and miR-7975, RNA extracted from the 30 MDMA users and 30 control samples (including the eight RNA samples used in the microarray) were studied using qRT-PCR. Complementary DNA (cDNA) synthesis was studied by using a “TaqMan MicroRNA Reverse Transcription Kit”, following the manufacturer’s protocol (Applied Biosystems, Foster City, CA). For each selected miRNA, TaqMan assays were purchased from Applied Biosystems, Foster City, CA. small-nucleolar RNA (snoRNA) RNU43 was used for normalization of miRNA expression analyses. (*mir7975 5’ AUCCUAGUCACGGCACCA-3’, mir1202 5’- GUGCCAGCUGCAGUGGGGGAG-3*).

Quantitative RT-PCR was performed using Universal Master Mix (Applied Biosystems) in a Roche LightCycler 480-II real-time thermal cycler (Roche, Switzerland, Basel). The reactions were applied in the following conditions: one cycle of 95 °C for 5 min followed by 40 cycles of 95 °C for 10 s, 60 °C for 20 s, and 72 °C for 25 s. Each was performed in triplicate. The relative quantification analysis was performed by the delta-delta-Ct method (2-ΔΔCT) as described previously (26).

*Statistical analysis*

Data were plotted as mean ± standard error and two-sided Student’s tests were used for statistical analysis of the qRT-PCR. A two-sided p-value of 0.05 or below was considered statistically significant. Receiver operating characteristic (ROC) curves were plotted using SPSS (Statistical Package for the Social Sciences) to see the power of control and validated miRNAs to differentiate from the MDMA samples. For ROC analysis, logistic regression was conducted and the predicted probabilities were calculated for each miRNA for the VTA and NAc regions of brain. The area under the curve (AUC) was calculated with a 95% confidence interval and accepted as significantly different from 0.5 when the p-value was greater than 0.5, meaning that the logistic regression classifies the group significantly better than by chance.

## Results

In this study, we conducted miRNA profiling of the NAc and VTA regions of subjects who had used MDMA and control subjects. A total of 120 postmortem human brain tissues (30 from VTA, 30 from NAc and their matched controls) were obtained from the Istanbul Morgue Department of the Council of Forensic Medicine (Turkey). All of the included cases had been part of the Turkish population. The samples were screened for the presence of MDMA by the Istanbul Toxicology Department of the Council of Forensic Medicine (Turkey). Clinical and toxicology information about the postmortem MDMA and control individuals are provided in Table 1.

In every case, toxicology reports were reviewed and the absence or presence of MDMA was documented. MDMA concentrations ranged from 197.6 to 2017.8 ng/mL, with a median range of 554.75 ng/mL in postmortem blood samples. In the 30 MDMA positive cases, no other drugs of abuse were detected. The mean age of the MDMA user group was 27.96 ± 9.45, whereas the control group had an average age of 29.76 ± 10.57. Brain pH level changed from 5.56 to 6.98 in the MDMA group and 6.1 to 6.93 in the control group. No significant difference was found in PMI and brain pH levels between the two groups. Likewise, there was no difference between the RIN values of RNA extracted from the VTA and NAc regions of the MDMA users and the control group. Information on the subjects, including gender, age, PMI, RIN, and brain pH levels are summarized in Table 2.

**Figure 1:**
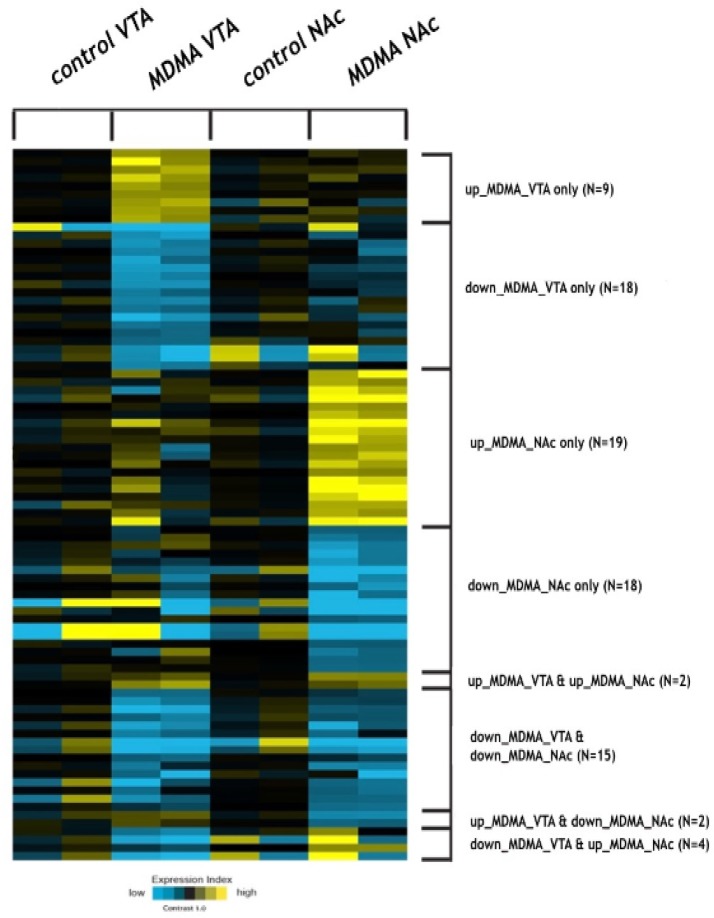
Heat-map illustration of deregulated miRNAs in VTA with MDMA samples vs VTA control samples and NAc with MDMA vs NAc control postmortem human brain tissues

Comparison of the miRNA expression profiles of the NAc and VTA brain regions of the postmortem human brain tissues from four of the MDMA users and four of the control samples was carried out using microarray analysis. According to the quantile normalized data, we found 41 significant deregulated miRNAs in the VTA regions, and 46 significant deregulated miRNAS in the NAc regions of both the MDMA-using group and the control group, with fold >1.4 between the two groups in both regions. A heat map illustration of differentially expressed miRNAs is presented in Figure 1.

**Figure 2:**
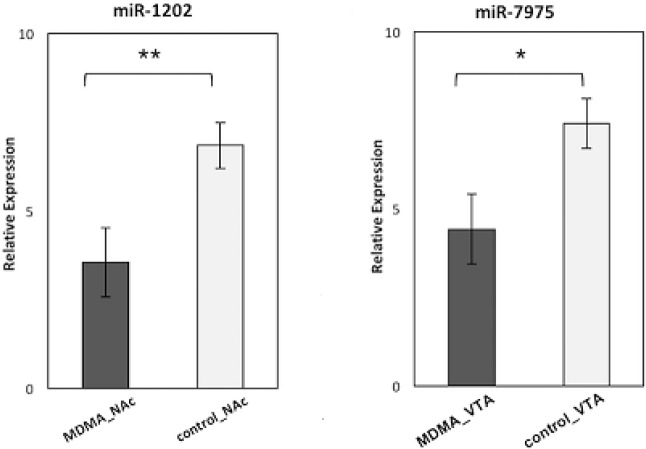
Relative expression levels of miR-1202 in 30 NAc with MDMA samples compared to 30 NAc control samples (*p *= 0.008), miR-7975 in 30 VTA with MDMA samples compared to 30 VTA control samples (*p *= 0.01).

Among the significantly differentially regulated miRNAs, miR-7975 was found to be down-regulated in only the VTA regions of the MDMA samples and miR-1202 to be down-regulated in only the NAc regions of the MDMA samples. Hence, miR-7975 and miR-1202 were selected, following a detailed literature search, for further qRT-PCR validation experiments. The qRT-PCR results confirmed that the expression of miR-7975 was significantly lower in the VTA region of the MDMA users than in that of the control samples. miR-1202 was found to have significantly reduced expression in the NAc region of the MDMA users compared to the control samples (Figure 2).

To test the power of miR-1202 and miR-7975 for distinguishing MDMA specimens from control samples, ROC were plotted. The values showed that miR-1202 (MDMA NAc vs control NAc) and miR-7975 (MDMA VTA vs control VTA) had an area under the curve (AUC) of 0.822, and 0.778, respectively (Figure 3). The results demonstrate their sufficiency to distinguish MDMA specimens from control specimens.

We also used three different miRNA target prediction databases (TargetScan 7.0 (27), mirDB (28) and miRWalk 2.0 (29)) to identify putative target genes and how they are affected by miR-1202 and miR-7975. Our bioinformatics studies have demonstrated that among the putative targets of mir-1202, *GRM4 *(30)*, GLP1 *(31)*, CTNN1 *(32), *GRIN1, GRIN2A, GRIN2B, SLC38, *and *GRIK3 *(33) were considerable candidates because their functions are associated with drug addiction or glutamatergic neurotransmission. In addition, human miRNA expression databases show that miR-1202 is highly expressed in the brain compared to other tissues (34, 35). This result indicates that miR-1202 may play an important regulatory role in its target genes in drug addiction. The analysis revealed that potential targets of miR-7975 – *PDE5A *(36), *SLC6A6, SLC6A9 *(37)*, CALN1 *(38)*, GDNF *(39)*, TMOD2, CHRNB2 *(40) – play a role in several drugs of abuse which affect neurotransmitter processes.

**Table 1:** Clinical information for the 60 postmortem brains included in this study

		**Clinical Diagnosis**		**Age**		**Gender**		**Cause of Death**		**MDMA in Blood [ng/mL]**		**PMI** **[hours]**		**pH**	
**1**		Control		31		M		Road traffic accident		N/A		17		6.13	
**2**		Control		49		M		Cardiac arrest		N/A		16		6.46	
**3**		Control		38		F		Acute coronary thrombosis		N/A		20		6.32	
**4**		Control		32		F		Work accident		N/A		17		6.12	
**5**		Control		32		M		Work accident		N/A		14		6.67	
**6**		Control		25		M		Cardiac arrest		N/A		18		6.84	
**7**		Control		23		M		Gunshot wounds		N/A		23		6.9	
**8**		Control		18		M		Road traffic accident		N/A		19		6.41	
**9**		Control		17		F		Road traffic accident		N/A		9		6.65	
**10**		Control		19		M		Road traffic accident		N/A		23		6.48	
**11**		Control		48		M		Gunshot wound		N/A		18		6.59	
**12**		Control		38		M		Road traffic accident		N/A		24		6.64	
**13**		Control		52		F		Atherosclerotic heart disease		N/A		19		6.4	
**14**		Control		19		M		Road traffic accident		N/A		8		6.7	
**15**		Control		21		M		Road traffic accident		N/A		15		6.54	
**16**		Control		24		M		Road traffic accident		N/A		19		6.43	
**17**		Control		26		M		Gunshot wound		N/A		23		6.1	
**18**		Control		30		F		Road traffic accident		N/A		13		6.14	
**19**		Control		37		M		Hypertensive heart disease		N/A		13		6.32	
**20**		Control		18		M		Road traffic accident		N/A		10		6.8	
**21**		Control		35		F		Hypertensive heart disease		N/A		17		6.46	
**22**		Control		41		M		Cardiac arrest		N/A		12		6.93	
**23**		Control		28		F		Road traffic accident		N/A		8		6.43	
**24**		Control		15		M		Road traffic accident		N/A		22		6.41	
**25**		Control		36		F		Gunshot wounds		N/A		14		6.52	
**26**		Control		32		M		Work accident		N/A		22		6.36	
**27**		Control		47		M		Cardiac arrest		N/A		20		6.84	
**28**		Control		18		F		Work accident		N/A		18		6.48	
**29**		Control		24		F		Gunshot wounds		N/A		15		6.76	
**30**		Control		20		F		Work accident		N/A		11		6.85	
**31**		MDMA user		32		M		Death due to MDMA intoxication		1523.6		23		6.64	
**32**		MDMA user		28		M		Death due to MDMA intoxication		606.8		15		6.88	
**33**		MDMA user		33		M		Death due to MDMA intoxication		1119.2		12		6.66	
**34**		MDMA user		19		M		Road traffic accident		318.6		9		6.36	
**35**		MDMA user		18		F		Hyperpyrexia		402.3		15		6.66	
**36**		MDMA user		32		F		Gunshot wounds		207.1		12		6.48	
**37**		MDMA user		47		M		Road traffic accident		276.9		22		6.68	
**38**		MDMA user		22		F		Gunshot wound		401.3		18		6.98	
**39**		MDMA user		30		M		Death due to MDMA intoxication		1023.6		12		6.87	
**40**		MDMA user		31		F		Death due to MDMA intoxication		756.3		13		6.63	
**41**		MDMA user		46		M		Death due to MDMA intoxication		2017.8		15		6.55	
**42**		MDMA user		16		M		Gunshot wounds		269.3		22		6.7	
**43**		MDMA user		20		F		Road traffic accident		252.8		21		6.8	
**44**		MDMA user		17		F		Fall from high		197.6		17		6.94	
**45**		MDMA user		35		F		Death due to MDMA intoxication		569.3		23		6.4	
**46**		MDMA user		36		M		Gunshot wounds		262.7		14		5.92	
**47**		MDMA user		20		F		Death due to MDMA intoxication		669.7		19		6.62	
**48**		MDMA user		35		M		Death due to MDMA intoxication		1324.6		23		6.9	
**49**		MDMA user		27		M		Death due to MDMA intoxication		540.2		24		6.14	
**50**		MDMA user		20		F		Death due to MDMA intoxication		524.6		22		6.42	
**51**		MDMA user		16		M		Death due to MDMA intoxication		825.6		14		6.62	
**52**		MDMA user		47		M		Cardiac arrest		450.6		14		6.31	
**53**		MDMA user		33		M		Death due to MDMA intoxication		895.3		16		6.7	
**54**		MDMA user		21		F		Death due to MDMA intoxication		908.7		15		6.8	
**55**		MDMA user		17		M		Death due to MDMA intoxication		654.4		12		6.43	
**56**		MDMA user		20		F		Road traffic accident		306.8		13		6.6	
**57**		MDMA user		24		M		Gunshot wound		379.5		18		5.56	
**58**		MDMA user		42		F		Death due to MDMA intoxication		648.7		24		6.86	
**59**		MDMA user		31		M		Road traffic accident		295.3		17		6.6	
**60**		MDMA user		24		M		Death due to MDMA intoxication		852.7		19		6.39	

**Table 2:** The characteristics of MDMA and control samples that are involved in the study

	**Control ** ***(N=30)***	**MDMA ** ***(N=30)***	***p *** **value**
**Sex**	19 M/11 F	18 M/12 F	-
**Age**	29.76 ±10.57	27.96±9.45	0.48
**Brain pH**	6.52±0.23	6.57±0.30	0.50
**RIN (VTA)**	6.38±0.28	6.47±0.34	0.25
**RIN (NAc)**	6.76±0.35	6.51±0.72	0.34
**PMI**	17.01±4.34	16.57±4.65	0.64

**P < 0.01, *P < 0.05; *t*-test.

## Discussion

MDMA use has increased dramatically among adolescents despite growing evidence of its potential harmful effects (16). Although various therapies have been developed to treat MDMA abusers, most of the treatment options available have proved insufficiently effective. Thus, there is an urgent need to identify the underlying mechanisms of MDMA-seeking behavior and to develop effective therapeutic approaches to MDMA abuse.

miRNAs are among the most promising potential therapeutic targets for MDMA because of their ability to be ‘key organizers’’ of gene regulation and expression. There are now several reports that show evidence of the correlation among drug addiction and miRNA regulation (22, 41). 

Latest studies have defined changes in aberrant miRNA expression profiles detected with drug consumption, along with drug induced neuroplasticity in the VTA and NAc, as well as other regions of the mesocorticolimbic DA system (21, 42, 43). Although a number of comprehensive analyses of several drug abuse miRNA expressions in animal models and human postmortem brains have been published over the years, profiling studies of differentially expressed miRNAs have not been conducted in connection with human MDMA seeking until now.

In this experiment, we researched the role of miRNAs in searching for therapeutic targets in MDMA users by examining post-mortem human brain tissues using microarray profiling of miRNA expression levels. 

Acting on the miRNA microarray levels results, we elective miR-1202 and miR-7975 the best highly dysregulated miRNAs that were altered expression in postmortem brain tissues. We validated the down-regulation of miR-7975 in 30 VTA regions from MDMA users and the down-regulation of miR-1202 in 30 NAc regions, with MDMA users’ brain tissues compared with matched control postmortem brain tissues.

MiR-1202, is a neuron system-enriched miRNA that has been shown to have a key role in cognitive processes, and act through the modulation of the glutamatergic system (30, 44). Moreover, previous research has demonstrated that a decreased miR-1202 expression level was found to negatively correlate with *GRM4*, also detected in the postmortem brain samples. GRM4 is expressed through the brain and neuronal system which is involved in glutamatergic, dopaminergic, GABAergic, and serotonergic neurotransmission processes. Moreover, higher *GRM4* protein expression has beforehand been reported to be incorporated with major depression and connectivity in brain activity (30). 

Other studies have demonstrated that GLP1, also known as EMHT1, targets the miR-1202 gene, and plays a role in memory consolidation and retrieval in maintenance mode (45). In addition, the findings showed that alcohol, amphetamine, cocaine, and nicotine are modulated by* GLP1 *in brain stimulation and reward processes. Researchers suggest that *GLP1* analogues could be used in the treatment of drug dependence. It has been suggested that a down-regulated miR-1202 effect is mediated by the suppression of the target gene *GLP1,* which fuels drug addiction. (31). According to these results and those of our own labor, miR-1202 may be important as a potential therapy for MDMA abuse. In this study, we further highlighted miR-7975, whose functions have not yet been studied. Ron* et al* have shown that one target gene of miR-7975 is *GDNF*, which has a key role as a negative regulator of drug of abuse like cocaine and alcohol (39). Moreover, *TMOD2, *which is another target of mir-7975, has been previously shown to be involved in neurotransmitter discharge and synaptic plasticity in the adult brain (40). Every single microRNAs is able to regulate several hundred mRNAs; therefore, many other predicted mRNA targets may also subscribe to phenotypes consolidated with miR -1202 and miR-7975 deregulation by MDMA use. Therefore, altering the level of these miRNAs can regulate the differential expression of multiple genes, in replication to the MDMA toxicity, which can result in the molecular conformation that leads to abuse of drug. 

## Conclusion

To the best of our knowledge, this is the first study in literature of a consistent miRNA dysregulation in MDMA-affected postmortem human brain tissues. Overall, our findings show that miR-1202 and miR-7975 are differentially expressed in MDMA-positive individuals, and these miRNAs can serve as potential biomarkers for predicting MDMA abuse and treatment response. Predicted miRNAs such as mir-1202 and miR-7975, have been linked in the mechanisms of abuse of drug, and forward studies can be highly important to developing preventive strategies and fresh therapeutic interventions for MDMA abuse.
